# Comparison of fecal culture and F57 real-time polymerase chain reaction for the detection of *Mycobacterium avium* subspecies *paratuberculosis* in Swiss cattle herds with a history of paratuberculosis

**DOI:** 10.1186/s13028-014-0068-9

**Published:** 2014-10-10

**Authors:** Selina M Keller, Roger Stephan, Rahel Kuenzler, Mireille Meylan, Max M Wittenbrink

**Affiliations:** Institute of Veterinary Bacteriology, Vetsuisse Faculty, University of Zurich, Winterthurerstrasse 270, CH-8057 Zurich, Switzerland; Institute for Food Safety and Hygiene, Vetsuisse Faculty, University of Zurich, Winterthurerstrasse 270, 8057 Zurich, Switzerland; Clinic for Ruminants, Vetsuisse Faculty, University of Berne, Bremgartenstrasse 109a, 3012 Berne, Switzerland

**Keywords:** *Mycobacterium avium* subspecies *paratuberculosis*, Cattle, Feces, Culture, PCR

## Abstract

**Background:**

Bovine paratuberculosis is an incurable chronic granulomatous enteritis caused by *Mycobacterium avium* subspecies *paratuberculosis* (MAP). The prevalence of MAP in the Swiss cattle population is hard to estimate, since only a few cases of clinical paratuberculosis are reported to the Swiss Federal Food Safety and Veterinary Office each year.

Fecal samples from 1,339 cattle (855 animals from 12 dairy herds, 484 animals from 11 suckling cow herds, all herds with a history of sporadic paratuberculosis) were investigated by culture and real-time polymerase chain reaction (PCR) for shedding of MAP.

**Results:**

By culture, MAP was detected in 62 of 445 fecal pools (13.9%), whereas PCR detected MAP in 9 of 445 pools (2.0%). All 186 samples of the 62 culture-positive pools were reanalyzed individually. By culture, MAP was grown from 59 individual samples (31.7%), whereas PCR detected MAP in 12 individual samples (6.5%), all of which came from animals showing symptoms of paratuberculosis during the study. Overall, MAP was detected in 10 out of 12 dairy herds (83.3%) and in 8 out of 11 suckling cow herds (72.7%).

**Conclusions:**

There is a serious clinically inapparent MAP reservoir in the Swiss cattle population. PCR cannot replace culture to identify individual MAP shedders but is suitable to identify MAP-infected herds, given that the amount of MAP shed in feces is increasing in diseased animals or in animals in the phase of transition to clinical disease.

## Background

*Mycobacterium avium* subspecies *paratuberculosis* (MAP) is the etiological agent of paratuberculosis (Johne’s disease), an incurable chronic granulomatous enteritis primarily affecting wild and domestic ruminants. MAP-infected cattle may have implications for public health, because the agent is putatively linked to a human chronic granulomatous ileitis described as Crohn’s disease [1]. In the dairy industry, paratuberculosis has been globally recognized as an important cause of morbidity and hence of significant economic loss [2]. In European countries, the prevalence of paratuberculosis amongst cattle is approximately 20% [3]. In Switzerland, the rate is considerably lower, i.e. 10% on average [4,5]. However, the significance of MAP as a pathogen in the Swiss cattle population is hard to estimate. On average, only 15 cases of clinical bovine paratuberculosis-out of an overall cattle population of approximately 1.6 million animals-are reported to the Swiss Federal Food Safety and Veterinary Office (FSVO) each year.

Bearing this in mind, systematic analyses of the prevalence of MAP in Swiss dairy cattle are indicated. Paratuberculosis is mainly diagnosed by means of direct confirmation of MAP in feces, either by culture or by targeting specific MAP gene sequences with polymerase chain reaction (PCR). The sensitivity of both culture and PCR are dependent on the stage of infection of individual animals. Irregular fecal shedding of (usually few) MAP cells occurs during the preclinical stage, whereas high numbers of MAP are shed during the late clinical stage [2]. Culture methods, although with an estimated sensitivity of maximally 60% not ideal, are considered the reference diagnostic tests for the direct detection of MAP in fecal samples [2,6]. PCR is often examined as an alternative to bacterial culture but is generally considered less sensitive than culture [2]. In comparison to culture as the reference method, a correlation between MAP numbers excreted in feces and the detection rate by PCR is evident: With so-called heavy shedders, PCR has a sensitivity of 80 to 100%, but PCR is not effective for the detection of cattle shedding low numbers of MAP [7-9].

In order to estimate the prevalence of MAP in the Swiss cattle population, we analyzed fecal samples of 1,339 cattle from herds with a history of sporadic paratuberculosis. We do not know how often the different stages of infection with MAP occur in indigenous cattle. Therefore, we conducted a systematic comparison of conventional culture and real-time PCR. This should prove which method provides a higher likelihood for the direct detection of MAP in fecal specimens from non-clinical cattle from indigenous herds with a history of sporadic paratuberculosis.

## Methods

### Animals and samples

The present study involved 1,339 cattle from 23 herds, i.e. 855 animals (including 7 bulls) from 12 dairy herds and 484 animals (including 22 bulls) from 11 suckling cow herds. Herd sizes ranged from 11 to 130 animals. The average age in the dairy herds was 4.2 years (minimum: 10 months, maximum: 13 years), and in the suckling cow herds it was 4.8 years (minimum: 11 months, maximum: 17 years). During the last 5 years, the FSVO registration office of notifiable diseases was informed of at least one animal per herd with confirmed paratuberculosis.

Fecal samples were collected over a period of 5 months, i.e. from February to July 2011. Samples were taken from the animals’ rectum by glove and without lubricant, subsequently chilled and then stored at −20°C until testing (for a maximum period of 3 months). At the time of sampling, all animals were clinically healthy, except for three cows presenting symptoms of paratuberculosis (2 cows from suckling cow herds; one cow from a dairy herd). Another 14 animals from 9 herds (7 animals from 3 suckling cow herds; 7 animals from 6 dairy herds) developed symptoms indicative of paratuberculosis (weight loss, reduced milk yield, intermittent diarrhea) without symptoms of general disease during an average time period of 7 months after sampling (minimum: 1 month, maximum: 16 months).

### Pooling and decontamination procedure

Fecal samples were assayed in batches according to their sampling date. Individual samples were pooled solely within the same herd. Thus, most pools consisted of three individual samples, with two or four samples also being possible. Pooling was performed by weighing 2.0 g of feces from each of the 3 animals into a sterile 50 ml polycarbonate screw capped tube (Becton Dickinson, Basel, Switzerland). 8.0 ml of sterile water and glass beads (4 mm Ø, Faust, Schaffhausen, Switzerland) were added to improve homogenization by vigorous vortexing. Subsequently, two 3.0 ml aliquots of the homogenate were subjected to the decontamination procedure according to Beerwerth [10]. Briefly, each 3 ml aliquot was homogenized in 40 ml of 4.0% NaOH (Roth, Karlsruhe, Germany) with glass beads by repeated agitation on a vortexer, shaken for a further 8 min on a horizontal shaker and then allowed to stand for 2 min in order for gross particles to settle. The supernatants were then pooled. Upon centrifugation (3000 × g, 15 min, 20°C) the pellet was resuspended by thorough repeated agitation on a vortexer (in 20 ml of 5% oxalic acid) and then shaken for 15 min on a horizontal shaker. The suspension was centrifuged as described above. The pellet was then suspended once again in 6 ml of sterile saline (0.15 M NaCl) and used as inoculum.

### Culture

Loewenstein-Jensen (LJ) medium containing 2.0 mg/l mycobactin J (Enclit, Oelzschau, Germany) and Herrold’s Egg Yolk Agar (HEYA) with 2.0 mg/l mycobactin J, 50 μg/ml vancomycin, 50 μg/ml of nalidixic acid, and 50 μg/ml of amphotericin B (Becton Dickinson, Basel, Switzerland) were used as culture media. 200 μl aliquots of the inoculum were transferred to each of two slants of LJ and HEYA, respectively. The inoculated tubes were left to stand for 1 h at ambient temperature so that the agar surface was horizontal, thus permitting maximum absorption of the inoculum on to the agar surface [11]. Cultures were incubated at 37°C for 16 weeks and inspected for growth of MAP at biweekly intervals. MAP was preliminarily diagnosed on the basis of colony morphology (small, smooth to slightly rough, opaque to whitish colonies). All presumptive positive colonies were picked up with sterile swabs and transferred into 3 ml of Middlebrook 7H9 broth, supplemented with ADC-enrichment (Becton Dickinson, Basel, Switzerland) and 2.0 mg/l mycobactin J (Synbiotics Europe, Lyon, France). Broth cultures were incubated for at least one week at 37°C and microscopically tested for acid-fast staining bacteria, following Ziehl-Neelsen staining [12]. All cultures with mycobacterial growth confirmed by microscopy were subjected to a F57-PCR specific for MAP. Fecal samples of all pools testing positive for MAP were reanalyzed individually by culture and PCR in order to identify the MAP shedders.

### Preparation of genomic DNA from fecal samples

A DNA extraction method described in detail previously was used to isolate genomic DNA from bovine feces [13,14]. Briefly, approximately 500 to 700 mg from each fecal specimen were resuspended in 2.0 ml of 0.15 M phosphate buffered saline (PBS, 136.9 mM NaCl, 1.46 mM KH2PO4, 8.1 mM Na2HPO4 × 2H2O, 2.7 mM KCl, pH 7.4). 100 μL of the sample suspensions were mixed with 130 μL lysis buffer and 20 μL of Proteinase K provided in the MaGNA Pure DNA Isolation Kit III (Roche Molecular Diagnostics, Penzberg, Germany) and incubated for 30 min at 65°C and 10 min at 95°C. The mixtures were transferred onto a bead-beating matrix in MagNA lyser tubes (Roche). A mechanical lysis step consisting of 60 s at 6,500 rpm followed by 60 s on a cooling block held at 4°C was performed three times on the samples. 100 μL of sample lysates were transferred to the MagNA Pure LC instrument sample cartridge. DNA was automatically isolated from these samples using the MagNA Pure LC instrument and in accordance with the protocol of the MagNA Pure LC DNA Isolation Kit III. Purified DNA templates were eluted in 100 μL of elution buffer provided in the DNA isolation kit. A 2 μL aliquot from the final eluate was used as a template for PCR.

### Real-time PCR

A real-time PCR protocol for analysis of bovine milk and feces targeting a 254-bp fragment within the MAP-specific F57 sequence and a 257-bp internal control (IC) template was used [13,14]. PCR reactions were carried out in the LightCycler 2.0 instrument (Roche Molecular Diagnostics) in a total reaction volume of 20 μl in glass capillary tubes. The reaction mixture contained 1 × LightCyclerFaststart DNA master plus hybridization probes mix (Roche Molecular Diagnostics), 1 μM of each primer (MAP f57p1, MAP f57p2), 200 nM of each LC probe (MAP f57-3′Fluo, MAP f57-5′LC Red 640, puC19-5′LC Red 705), 2 μl DNA template (original concentration of the extracted DNA), and 20 DNA copies of IC template. The amplification consisted of an initial pre-incubation step at 95°C for 10 min to activate the DNA polymerase, followed by 45 cycles of 95°C for 10 s, 56°C for 20 s, and 72°C for 18 s. The fluorescence signals corresponding to the F57 sequence target and the IC template amplification were monitored during the 56°C annealing step in the LC Red 640 nm and LC Red 705 detection channels of the LightCycler 2.0 instrument, respectively.

## Results

By culture, MAP was detected in 62 of the 445 fecal pools (13.9%). In detail, 48 isolates (77.4%) were grown only on HEYA, 2 (3.2%) only on LJ, and 12 (19.4%) on both HEYA and LJ. MAP was grown within 9 to 11 weeks of incubation. Of the 1,780 cultures (445 pools, 4 agar slants per pool), 16 (0.9%) were heavily contaminated by constituents of the bovine fecal flora and were thus excluded. However, investigation of the pools in question could be finalized, since there was only one medium tube per pool that could not be evaluated. Using F57 real-time PCR, MAP was detected directly in 9 of the 445 pools (2.0%).

To identify the MAP shedders, all 186 samples of the 62 culture-positive pools were reanalyzed individually by culture and by PCR. MAP was isolated from 59 of the 186 individual samples (31.7%), representing 50 of the 62 MAP-positive pools (80.6%). In detail, we identified 43 pools with 1 positive sample, 5 pools with 2 positive samples, and 2 pools with 3 positive samples (Σ 59 MAP isolates). By PCR, MAP was detected directly in 12 samples (6.5%) from 11 pools (10 pools with 1 positive sample; 1 pool with 2 positive samples). All PCR-positive samples also tested positive by culture. None of the culture-negative samples tested positive by PCR. Overall, MAP was detected in 10 out of 12 dairy herds (83.3%) as well as in 8 out of 11 suckling cow herds (72.7%).

Out of the 1,339 cattle a total of 17 animals showed symptoms of paratuberculosis either when the samples were taken (n =3), or over an average duration of 7 months after sampling (re. Methods). By culture, MAP was detected in fecal samples of all 17 animals. PCR detected MAP in fecal samples from 12 of the 17 animals (70.6%).

## Discussion

In the present study, we investigated a total of 1,336 fecal specimens from predominantly healthy cattle belonging to herds with a history of sporadic paratuberculosis. This sample collection provides a valid basis for a comparison of PCR and culture to detect MAP in feces from cattle without clinical signs of paratuberculosis. The cultural assay is significantly more sensitive than the F57 real-time PCR for this specific purpose. Cultivation of MAP proved to be successful in 62 fecal pools (13.9%) as well as in 59 of the 186 individual fecal samples (31.7%), whereas only 9 fecal pools (2.0%) and 12 individual fecal samples (6.5%) tested positive with PCR. It is noteworthy that only 50 of the 62 MAP-positive pools (80.6%) were confirmed by isolation of MAP from the individual samples. Culturing a single fecal specimen has a sensitivity of maximally 60% [2]. Thus, it is not unlikely that repeated analysis of a paucibacillary sample with inhomogeneously distributed MAP cells reveals a negative result. All in all, our finding of a considerably higher analytical sensitivity of culture for samples from non-clinical animals is in agreement with the literature [7-9].

PCR methods for MAP are considered less sensitive than bacterial culture for the following reasons: In comparison to diseased cattle, MAP-infected cattle in the preclinical stage only shed small amounts of bacteria in their feces [7-9,15,16]. The chance of detecting such small bacterial counts is much more likely in culture, because a much larger inoculum can be tested than with PCR. According to our protocol, the inoculum for culture was equivalent to 336 mg of feces per pool or individual sample. This amount was inoculated onto four agar slants which were subsequently incubated for 16 weeks and examined for growth of MAP at least 8 times (every other week). By comparison, the template for our PCR consisted of a DNA extract equivalent to 0.24 mg feces per PCR reaction. Hence the aliquot subjected to culture was 1,400 times higher than the aliquot analyzed by PCR.

In preceding evaluation studies, our DNA extraction and PCR protocol revealed a reproducible limit of detection (LOD) of 7 fg purified genomic MAP-DNA (arithmetically 1.4 MAP-DNA copies) per μl PCR template, equivalent to a LOD of 3.9 × 10^3^ MAP cells per gram feces in our study [13,14]. In comparison, an inoculum for the *in vitro*-growth of MAP needs to contain 50 viable mycobacteria on average [17,18]. Therefore, for the cultural growth of MAP according to our protocol, a fecal sample must contain no less than 5.9 × 10^2^ of MAP per gram of feces, which is approximately one sixth of the MAP counts required for a positive PCR. The much higher rate of MAP detection in culture than with PCR can also be led back to the fact that we subcultivated all MAP-suspected colonies systematically. An average of two subcultures per sample was applied, taking into consideration all samples (445 pools, 186 individual samples). These considerations explain why culture is more suitable than PCR to identify animals with asymptomatic intestinal MAP infections and low bacterial shedding.

MAP culture is very labor-intensive and thus probably less feasible for routine diagnostics. The validity of PCR for monitoring bovine herds affected by paratuberculosis had to be assessed further. Our tests have proven that the state of MAP infection in herds can be determined by PCR quite clearly with animals upon the onset of paratuberculosis symptoms, because cattle shed larger amounts of bacteria in the clinical state of paratuberculosis (2,15,16). Since also non-clinical animals – or as in our study animals in the phase of transition to clinical disease – can shed MAP in large numbers, our PCR protocol should enable the rapid screening of herds for heavy fecal shedders, the detection of which is more critical for herd control [9]. Applying only PCR is problematic when dealing with herds where, as was the case in our study, mainly low shedders are to be expected. In our study, 6 out of 18 MAP-positive herds (30.0%) could not be detected by PCR. A recent paper refers to the fact that the systematic optimization of the DNA extraction may allow the sensitivity of the PCR to meet the sensitivity of culture [19]. However, it remains unaddressed if an approximately 8 times higher sensitivity of PCR in comparison to culture [20,21] is solely based on an optimized DNA extraction. In any case, this important technical aspect must be verified within the scope of further systematic studies.

In the literature, various approaches to grow MAP from bovine feces are described without any one standard being generally accepted. This applies, in particular, to the decontamination of fecal samples (maximum inactivation of fecal bacterial contaminants whilst preserving viability of MAP). We decided to ensure decontamination by means of NaOH and oxalic acid. This method has been described several times as being quite effective, because very few cultures are lost due to an overgrowth by contaminants from the fecal flora [4,10,11,22-26]. NaOH is mainly advantageous because an alkaline environment causes less damage to mycobacteria than an acidic environment [27,28]. According to our results, alkali-coupled oxalic acid treatment introduced in mycobacteriological diagnostics 83 years ago [29] leads to a considerably higher detection rate of mycobacteria from feces than other decontamination methods.

## Conclusions

Compared to a F57 real-time PCR, culture is more sensitive for the detection of fecal MAP shedding in non-clinical cases of bovine paratuberculosis. Thus, PCR cannot replace culture to detect individual non-clinical MAP shedders. However, our F57 real-time PCR is suitable to identify MAP-infected herds if single animals are shedding MAP in larger numbers. These cattle are highly contagious in terms of risk of transmission to susceptible cattle. In our study, all MAP-shedders identified directly by PCR showed symptoms of paratuberculosis either at the time of sampling or during an average time period of 7 months after sampling. In Switzerland, cattle herds with sporadic clinical cases of paratuberculosis pose a serious risk of MAP-transmission to other herds due to the considerable rate of non-clinical fecal shedders.
